# Health care resource use among patients with advanced non-small cell lung cancer: the PIvOTAL retrospective observational study

**DOI:** 10.1186/s12913-018-2946-8

**Published:** 2018-03-01

**Authors:** Dae Ho Lee, Hiroshi Isobe, Hubert Wirtz, Sabina Bandeira Aleixo, Phillip Parente, Filippo de Marinis, Min Huang, Ashwini Arunachalam, Smita Kothari, Xiting Cao, Nello Donnini, Ann-Marie Woodgate, Javier de Castro

**Affiliations:** 1 Asan Medical Center, Seoul, Republic of Korea; 2 KKR Sapporo Medical Center, Sapporo, Japan; 3 University of Leipzig, Leipzig, Germany; 4 Oncology Services, Hospital Evangelico, Cachoeiro de Itapemirim, Brazil; 5 Cancer Services, Box Hill Hospital, and Monash University, Victoria, Australia; 6 Thoracic Oncology Division, European Institute of Oncology (IEO), Milan, Italy; 7 Center for Observational and Real World Evidence (CORE), Merck & Co., Inc., North Wales, PA USA; 8 Center for Observational and Real World Evidence (CORE), Merck & Co., Inc., 2000 Galloping Hill Road, Kenilworth, NJ 07033 USA; 9 MSD Italia, Rome, Italy; 10 MSD Australia & New Zealand, Sydney, Australia; 11 Medical Oncology Service, Hospital Universitario La Paz (IDIPAZ), Madrid, Spain

**Keywords:** Emergency visits, Health care resource use, Hospitalizations, Non-small cell lung cancer, NSCLC, Outpatient visits

## Abstract

**Background:**

Data are scarce regarding real-world health care resource use (HCRU) for non-small cell lung cancer (NSCLC). An understanding of current clinical practices and HCRU is needed to provide a benchmark for rapidly evolving NSCLC management recommendations and therapeutic options. The objective of this study was to describe real-world HCRU for patients with advanced NSCLC.

**Methods:**

This multinational, retrospective chart review study was conducted at academic and community oncology sites in Italy, Spain, Germany, Australia, Japan, South Korea, Taiwan, and Brazil. Deidentified data were drawn from medical records of 1440 adults (≥18 years old) who initiated systemic therapy (2011 to mid-2013) for a new, confirmed diagnosis of advanced or metastatic (stage IIIB or IV) NSCLC. We summarized HCRU associated with first and subsequent lines of systemic therapy for advanced/metastatic NSCLC.

**Results:**

The proportion of patients who were hospitalized at least once varied by country from 24% in Italy to 81% in Japan during first-line therapy and from 22% in Italy to 84% in Japan during second-line therapy; overall hospitalization frequency was 2.5–11.1 per 100 patient-weeks, depending on country. Emergency visit frequency also varied among countries (overall from 0.3–5.9 per 100 patient-weeks), increasing consistently from first- through third-line therapy in each country. The outpatient setting was the most common setting of resource use. Most patients in the study had multiple outpatient visits in association with each line of therapy (overall from 21.1 to 59.0 outpatient visits per 100 patient-weeks, depending on country). The use of health care resources showed no regular pattern associated with results of tests for activating mutations of the epidermal growth factor receptor (*EGFR*) gene or anaplastic lymphoma kinase (*ALK*) gene rearrangements.

**Conclusions:**

HCRU varied across countries. These findings suggest differing approaches to the clinical management of advanced NSCLC among the eight countries. Comparative findings and an understanding of country-specific clinical practices can help to identify areas of need and guide future resource allocation for patients with advanced NSCLC. Further studies evaluating the costs associated with resource use are warranted.

**Electronic supplementary material:**

The online version of this article (10.1186/s12913-018-2946-8) contains supplementary material, which is available to authorized users.

## Background

Lung cancer was responsible for 1.6 million deaths globally in 2012 [1]. Non-small cell lung cancer (NSCLC) is the most common form, accounting for 80–85% of all cases of lung cancer [1–3]. Risk factors for developing NSCLC include tobacco smoking (the number one risk factor) and exposure to second-hand tobacco smoke, airborne carcinogens, radon gas, and cooking fumes [1, 4]. The diagnosis of NSCLC is often, indeed in up to 65% of cases, made in advanced stages when the tumor(s) are nonresectable because of local infiltration or distant metastasis (stages IIIB and IV) [5, 6]. Until recently, the recommended systemic therapy for patients with advanced (nonresectable) NSCLC has been platinum-based chemotherapy in first-line, followed by single agents, such as docetaxel or pemetrexed, for patients who fail to respond or who experience disease progression after chemotherapy [6]. Even with therapy, the 5-year survival rates for patients with advanced NSCLC are 5% or lower [4].

Fortunately the list of treatment options for advanced NSCLC has expanded rapidly in the past 15 years, with continued optimistic outlook for expansion, because of two major advances: the discovery of molecular biomarkers that identify improved response to targeted agents and the development of immunotherapies, which assist patients’ immune response in attacking their cancer [6–8]. Currently the two most commonly diagnosed targetable biomarkers, each of which are accompanied by improved potential for response to specific tyrosine kinase inhibitors (TKIs), are activating mutations of the epidermal growth factor receptor (*EGFR*) gene and anaplastic lymphoma kinase (*ALK*) gene rearrangements. Immunotherapies recently approved for treating advanced NSCLC include the programmed death-1 (PD-1) receptor inhibitors, nivolumab and pembrolizumab [9]. The place in therapy for these newer agents remains an area of active study.

Patients with NSCLC usually require high levels of health care resource use (HCRU) that includes diagnostic testing, treatments, frequent office visits, potential hospitalizations, as well as supportive care at home, in hospital, and in hospice. However, data are scarce regarding the real-world utilization of health care resources for NSCLC [10]. Clinical trial data have limited utility for assessing real-world HCRU both because key information, such as that regarding hospitalizations, may not be collected and because patients enrolled in clinical trials are not representative of those treated in real-world clinical practice [11]. The eligibility criteria for NSCLC clinical trials typically exclude patients with poor performance status, comorbidities, untreated brain metastases, or insufficient biopsy tissue to fully characterize their cancer. Therefore real-world data are important to understand HCRU in routine clinical practice and, as important, for use in economic modeling to support decision-making by providers and payers [12].

The PIvOTAL study (Global treatment **P**atterns, resource utilization and b**IO**marker **T**esting of **A**dvanced non-small cell **L**ung cancer) was a retrospective observational study designed to address the scarcity of real-world data regarding the treatment of patients with advanced/metastatic (Stage IIIB/IV) NSCLC in nine countries in different regions of the world. The primary objectives of the study were to examine treatment patterns, biopsy and predictive biomarker testing patterns, and associated HCRU for patients with advanced NSCLC. Here we report the HCRU findings from eight countries in the PIvOTAL study. The treatment pattern data for these eight countries have been previously published [13, 14], and the findings from Canada are being considered for future publication.

## Methods

### Data source and patients

This multinational, retrospective observational study was conducted at both academic and community oncology sites in Italy, Spain, Germany, Australia, Japan, South Korea, Taiwan, and Brazil. Detailed study methods have been published [13]. Electronic case report forms (eCRFs) were used to collect de-identified data abstracted from medical records of patients with stage IIIB/IV NSCLC. A single individual at each site was charged with data abstraction to maximize consistency, working under the supervision of a lead site investigator who was responsible for data review.

Eligible patients for study were adults (≥18 years old) who received systemic therapy for a new diagnosis between January 1, 2011, and July 1, 2013, of histopathologically confirmed advanced/metastatic NSCLC (stage IIIB or IV, confirmed by tissue biopsy or cytology). In addition, eligible patients had to have complete medical records from the time of diagnosis to the end of the study, or death, whichever occurred first. Patients who had presented with an earlier stage of NSCLC or who did not initiate systemic therapy for NSCLC were excluded, as were those who had other cancer or who were participating in a clinical trial.

To gather the most recent data, eligible patients were identified starting at the end of the eligibility period (July 1, 2013) and working backwards in time. (In Germany, the eligibility period was extended to July 1, 2014, to recruit adequate patient numbers.) The index date for each patient was defined as the start date of first-line NSCLC therapy. The follow-up period was defined as being until the initiation of first medical record abstraction in the patient’s country (in 2015 or 2016, depending on country), or until death, whichever occurred first [13].

The study protocol was approved by the appropriate institutional review board or independent ethics committee for each study site (Additional file 1). Informed consent was collected for patients from Italy and Spain who were alive at the time of data abstraction. In the other countries informed consent was not required for working with de-identified retrospective data.

### Outcome measures

The key outcome measures were weekly HCRU rates and total HCRU, by line of therapy, related to management of Stage IIIB/IV NSCLC from the date of diagnosis until the end of follow-up for each patient. We included hospitalizations, emergency and outpatient visits, imaging tests, biopsy-related procedures, and biomarker tests. In addition, we examined hospitalizations and outpatient visits according to the results of predictive biomarker testing and whether testing was performed, with focus on testing for sensitizing *EGFR* mutations and *ALK* gene rearrangements.

Hospitalization was defined as formal inpatient admittance to a hospital, either to a normal ward or intensive care unit, pursuant to an order for hospital admission by a physician or other qualified practitioner, for at least 24 h. An emergency visit was defined as a visit to a hospital-based emergency department. An outpatient visit was defined as including outpatient hospital, office-based, and outpatient infusion therapy visits; office-based visits could be to a specialist or primary care provider.

### Statistical analyses

This was an exploratory, descriptive study with no formal hypothesis testing. Data from patients’ medical records were abstracted and reported using summary statistics by country. Data were not pooled across countries because of different clinical practices in each country. Available data were reported for key variables; missing data were not imputed.

All HCRU data were summarized over the entire course of the study. The summarized data were then averaged for each line of therapy (LOT) to calculate HCRU per 100 patient-weeks using the following formulas:

- Weeks of follow-up period = (last visit/treatment stop date – index date + 1) / 7
- Weeks of LOT = (next LOT start date – LOT start date + 1) / 7
- Weekly HCRU rate during each LOT per 100 patient-weeks = 100*summarized HCRU data for the LOT / weeks of LOT

All analyses were carried out using SAS version 9.4 (SAS Institute, Cary, NC, USA).

## Results

### Patients and NSCLC therapy

We studied a total of 1440 patients with advanced NSCLC in eight countries, including 174 patients in Italy, 202 patients in Spain, 139 patients in Germany, 208 patients in Australia, 175 patients in Japan, 150 patients in South Korea, 217 patients in Taiwan, and 175 patients in Brazil. The median age of patients in each country ranged from 63 to 70 years. The distributions of other demographic and clinical characteristics of patients in the individual countries were similar with the exception of Taiwan. In all countries except Taiwan, the majority of patients were male (from 53% to 77%, but 48% male in Taiwan) and current or former smokers (65% to 88%, but 33% in Taiwan). Approximately three-quarters of patients had NSCLC of nonsquamous histology, and over 80% in each country presented with stage IV disease, as previously reported [13, 14].

All patients received first-line systemic therapy per study enrollment criteria, and subsequently from 46% (Germany) to 71% (Taiwan) in each country received second-line therapy, and from 17% (Brazil) to 42% (Taiwan) received third-line therapy. The majority of patients received platinum-based combinations for first-line therapy, most commonly carboplatin-paclitaxel, carboplatin-gemcitabine, or cisplatin-pemetrexed, except in Taiwan, where about half of patients received an EGFR TKI or ALK inhibitor. For second-line therapy, approximately half of patients received a single agent, most commonly docetaxel or pemetrexed, and approximately one-quarter received an EGFR TKI or ALK inhibitor. In general, treatment patterns by regimen category (platinum-based or non-platinum combination, single agent, EGFR/ALK TKI) varied only slightly by country, with the exception of first-line therapies in Taiwan [13, 14].

### Health care resource use by treatment line and regimen

The proportion of patients who were hospitalized varied greatly by country (Tables 1 and 2): namely, during first-line, from 24% in Italy to 81% in Japan and, during second-line, from 22% in Italy to 84% in Japan were hospitalized at least once. In Germany during both first- and second-line therapy, three-quarters of patients had a recorded hospitalization. The proportions of patients hospitalized were generally similar by treatment regimen, except in Taiwan, where those who received platinum-based regimens were more likely to be hospitalized than those who received a single agent or an EGFR/ALK TKI (Tables 1 and 2). Instead, outpatient visits in association with first- and second-line therapy were recorded for most patients, including 86% or more in each country, except in Germany during first-line therapy (69%; see Tables 1 and 2).

**Table 1:** Health care resource use by line of therapy and regimen in Italy, Spain, Germany, and Australia

	Italy			Spain			Germany			Australia		
	(*N* = 174)			(*N* = 202)			(*N* = 139)			(*N* = 208)		
Characteristic	*N* ^a^	Inpatient stay^b^	Outpatient visit	*N* ^a^	Inpatient stay	Outpatient visit	*N* ^a^	Inpatient stay	Outpatient visit	*N* ^a^	Inpatient stay	Outpatient visit
First-line therapy^c^	*149*	36 (24)	143 (96)	*130*	55 (42)	121 (93)	*81*	61 (75)	56 (69)	*184*	83 (45)	177 (96)
Platinum-based comb.	*119*	28 (24)	116 (98)	*107*	49 (46)	98 (92)	*73*	54 (74)	52 (71)	*156*	75 (48)	150 (96)
Non-platinum comb.	*0*	0	0	*3*	1 (33)	3 (100)	*0*	0	0	*0*	0	0
Single agent	*15*	2 (13)	13 (87)	*9*	1 (11)	9 (100)	*4*	3 (75)	1 (25)	*15*	4 (27)	14 (93)
EGFR/ALK TKI	*15*	6 (40)	14 (93)	*11*	4 (36)	11 (100)	*4*	4 (100)	3 (75)	*13*	4 (31)	13 (100)
Second-line therapy^c^	*89*	20 (22)	88 (99)	*68* ^d^	30 (44)	64 (94)	*37*	27 (73)	32 (87)	*118*	55 (47)	114 (97)
Platinum-based comb.	*12*	3 (25)	12 (100)	*14*	7 (50)	14 (100)	*11*	9 (82)	8 (73)	*21*	7 (33)	21 (100)
Non-platinum comb.	*0*	0	0	*6*	4 (67)	6 (100)	*2*	1 (50)	1 (50)	*1*	0	1 (100)
Single agent	*45*	12 (27)	44 (98)	*34*	9 (27)	33 (97)	*17*	11 (65)	16 (94)	*71*	34 (48)	69 (97)
EGFR/ALK TKI	*32*	5 (16)	32 (100)	*12*	9 (75)	9 (75)	*7*	6 (86)	7 (100)	*25*	14 (56)	23 (92)

Data are n (%) unless otherwise noted

^a^*N* represents patients with available HCRU data

^b^An inpatient stay was defined as one or more nights in a hospital or hospice

^c^The systemic therapy categories were defined as follows:

• Platinum-based combination: regimen with two or more anticancer therapies including carboplatin or cisplatin

• Non-platinum combination: regimen with two or more anticancer therapies not including carboplatin or cisplatin (can contain bevacizumab in combination with other non-platinum drug)

• Single agent: regimen of one anticancer drug that was not an EGFR or ALK tyrosine kinase inhibitor (TKI)

• EGFR/ALK TKI: monotherapy with anti-EGFR (erlotinib, gefitinib, afatinib) or anti-ALK agent (crizotinib, ceritinib)

• Other NSCLC anticancer agent: any other agent not included in the prior categories

^d^In Spain during second-line therapy, “Other” NSCLC agents were administered to 2 patients, one of whom had an inpatient stay and both of whom had outpatient visits

*ALK* anaplastic lymphoma kinase, *comb.* combination, *EGFR* epidermal growth factor receptor, *NSCLC* non-small cell lung cancer, *TKI* tyrosine kinase inhibitor

**Table 2:** Health care resource use by line of therapy and regimen in Japan, Korea, Taiwan, and Brazil

	Japan			Korea			Taiwan			Brazil		
	(*N* = 175)			(*N* = 150)			(*N* = 217)			(*N* = 175)		
Characteristic	*N* ^a^	Inpatient stay^b^	Outpatient visit	*N* ^a^	Inpatient stay	Outpatient visit	*N* ^a^	Inpatient stay	Outpatient visit	*N* ^a^	Inpatient stay	Outpatient visit
First-line therapy	*134*	109 (81)	117 (87)	*134*	83 (62)	130 (97)	*172*	94 (55)	149 (87)	*106*	33 (31)	94 (89)
Platinum-based comb.	*94*	84 (89)	79 (84)	*107*	67 (63)	103 (96)	*33*	26 (79)	24 (73)	*100*	30 (30)	89 (89)
Non-platinum comb.	*0*	0	0	*0*	0	0	*26*	17 (65)	24 (92)	*0*	0	0
Single agent	*10*	6 (60)	9 (90)	*3*	3 (100)	3 (100)	*29*	7 (24)	28 (97)	*2*	1 (50)	1 (50)
EGFR/ALK TKI	*30*	19 (63)	29 (97)	*24*	13 (54)	24 (100)	*84*	44 (52)	73 (87)	*4*	2 (50)	4 (100)
Second-line therapy	*89*	75 (84)	81 (91)	*93*	62 (67)	90 (97)	*131*	70 (53)	112 (86)	*63*	22 (35)	57 (90)
Platinum-based comb.	*22*	17 (77)	17 (77)	*11*	6 (55)	11 (100)	*35*	25 (71)	24 (69)	*7*	1 (14)	7 (100)
Non-platinum comb.	*2*	2 (100)	2 (100)	*3*	3 (100)	2 (67)	*30*	10 (33)	30 (100)	*1*	1 (100)	1 (100)
Single agent	*48*	42 (88)	47 (98)	*31*	21 (68)	31 (100)	*40*	22 (55)	36 (90)	*50*	18 (36)	44 (88)
EGFR/ALK TKI	*17*	14 (82)	15 (88)	*48*	32 (67)	46 (96)	*26*	13 (50)	22 (85)	*5*	2 (40)	5 (100)

Data are n (%) unless otherwise noted

^a^*N* represents patients with available HCRU data

^b^An inpatient stay was defined as one or more nights in a hospital or hospice

*ALK* anaplastic lymphoma kinase, *comb*. combination, *EGFR* epidermal growth factor receptor, *TKI* tyrosine kinase inhibitor

**Fig. 1:**
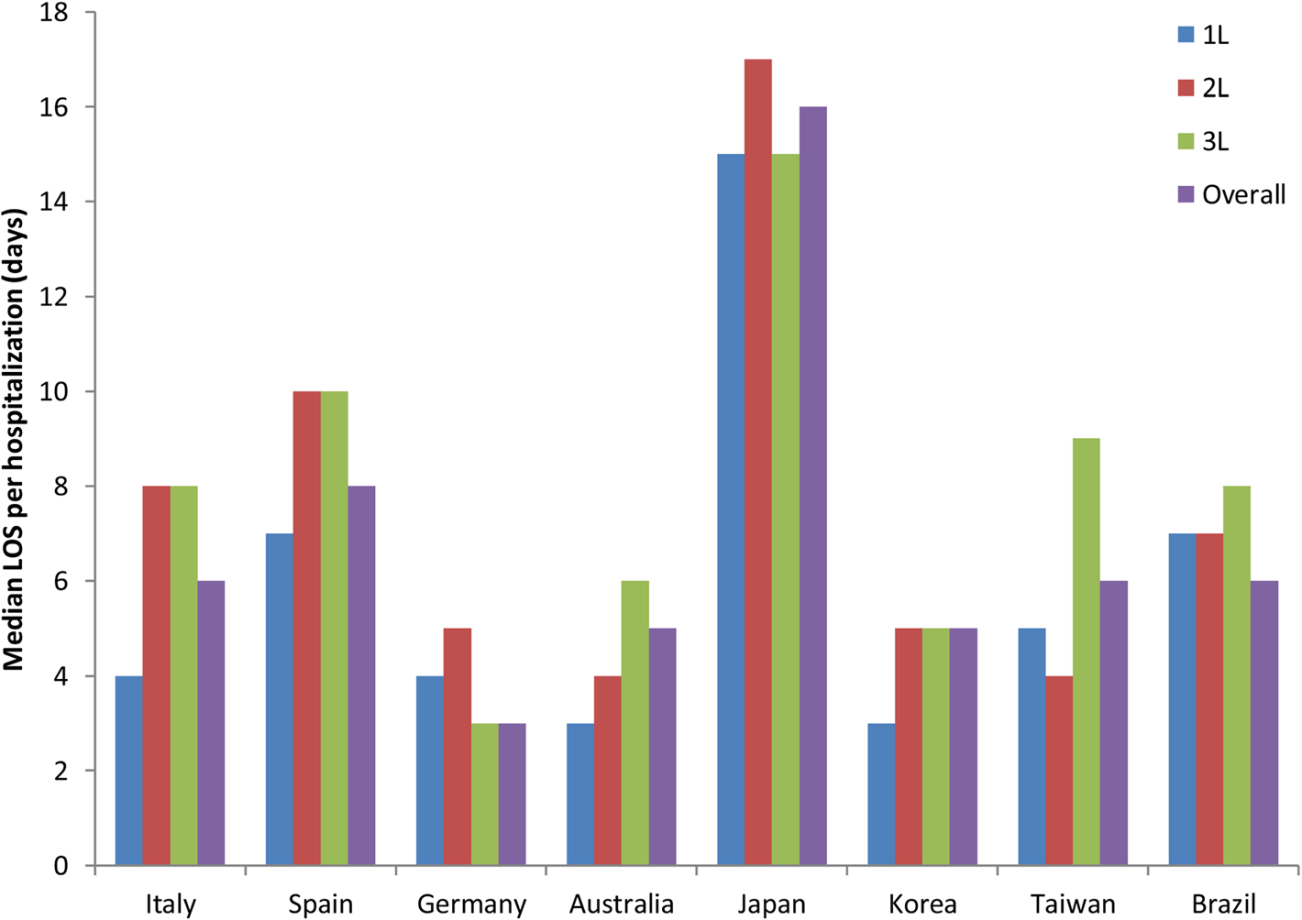
Median length of stay (LOS) per hospitalization by line of therapy and overall in each country. 1L, 2L, 3L, during first-, second-, and third-line therapy. Hospitalizations did not include hospice stays

**Fig. 2:**
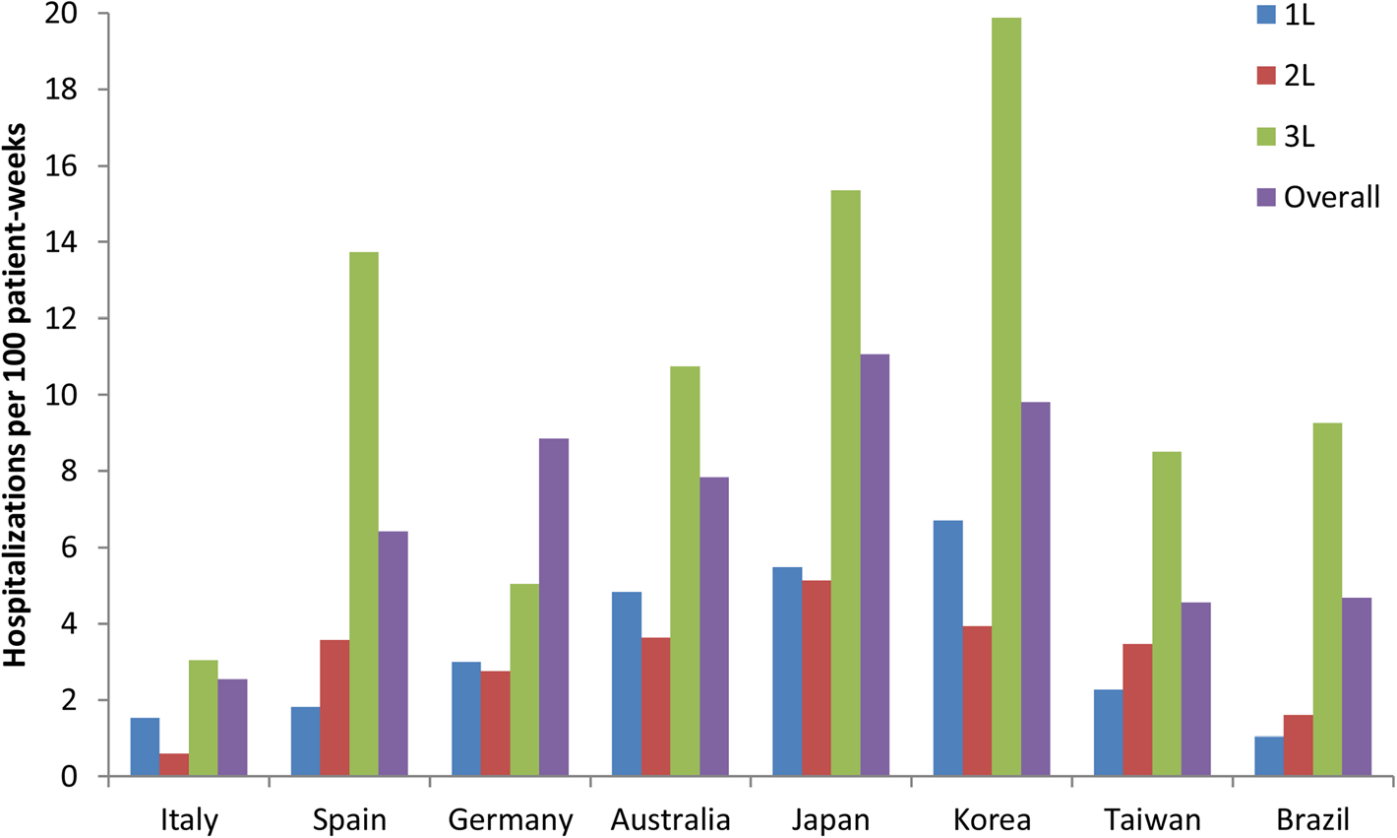
Number of hospitalizations per 100 patient-weeks by line of therapy and overall in each country. 1L, 2L, 3L, during first-, second-, and third-line therapy. Hospitalizations did not include hospice stays

Table 3 provides further detail on hospitalizations, emergency visits, and outpatient visits by country, including further detail regarding previously published HCRU data from Japan [14]. During first-line therapy, the length of each hospitalization and the total days of hospitalization per patient were greatest in Japan, with a median of 15 days per hospitalization and a median of 36 total days of hospitalization. In the other countries the median lengths were from 3 to 7 days per hospitalization and 8–14 total days during first-line therapy (Table 3). A similar pattern persisted during second-line therapy and overall, depicted in Fig. 1. The number of hospitalizations per 100 patient-weeks was highest in Korea in first-line and in Japan in second-line (Fig. 2). Admissions to hospice were few, recorded only in Spain, Australia, and Taiwan, with none in the other countries (Table 3).

**Table 3:** Hospitalizations and outpatient visits during first-line and second-line therapy

First-line therapy	Italy	Spain	Germany	Australia	Japan^c^	Korea	Taiwan	Brazil
	(*N* = 174)	(*N* = 202)	(*N* = 139)	(*N* = 208)	(*N* = 175)	(*N* = 150)	(*N* = 217)	(*N* = 175)
Admitted to hospital or hospice, n	36	55	61	83	109	83	94	33
Hospitalization	36 (100)	54 (98)	61 (100)	82 (99)	109 (100)	83 (100)	93 (99)	33 (100)
Hospice	0	2 (4)	0	6 (7)	0	0	1 (1)	0
Total no. hospitalizations^a^	75	112	140	284	242	251	200	42
No. hospitalizations per patient, median (range)	1 (1–7)	2 (0–7)	2 (1–7)	2 (0–34)	2 (1–10)	2 (1–10)	1 (0–9)	1 (1–3)
Total days of hospitalization per patient, mean (SD)	12.7 (14.1)	17.9 (17.0)	20.6 (29.2)	16.1 (24.4)	42.5 (28.5)	21.3 (29.1)	19.0 (19.3)	21.8 (26.5)
Median (range)	8 (1–77)	13 (1–100)	9 (1–160)	9 (1–199)	36 (3–146)	12 (1–178)	14 (2–139)	10 (1–90)
LOS per hospitalization (days)^b^								
Mean (SD)	6.1 (7.8)	8.7 (5.8)	9.0 (11.3)	4.6 (5.7)	19.1 (15.3)	7.1 (9.5)	8.9 (11.2)	17.0 (21.0)
Median (range)	4 (1–50)	7 (1–28)	4 (1–32)	3 (1–48)	15 (1–81)	3 (1–47)	5 (1–71)	7 (1–77)
No. hospitalizations per 100 pt-wk	1.53	1.81	3.00	4.83	5.49	6.70	2.27	1.03
Total no. emergency visits	6	153	5	73	9	64	125	64
No. emergency visits per 100 pt-wk	0.12	2.47	0.11	1.24	0.20	1.71	1.42	1.57
Patients with OP visits, n	143	119	56	177	117	130	149	94
OP visits, mean (SD)	7.6 (6.9)	9.0 (9.5)	8.7 (9.8)	11.5 (12.7)	14.2 (16.6)	10.6 (10.5)	14.0 (15.3)	7.0 (7.1)
OP visits, median (range)	5 (1–35)	7 (1–58)	4 (1–41)	7 (1–105)	9 (1–124)	8 (1–58)	10 (1–109)	5 (1–36)
No. OP visits per 100 pt-wk	22.02	17.33	10.43	34.74	37.65	36.94	23.57	16.24
Second-line therapy	*N* = 101	*N* = 96	*N* = 64	*N* = 128	*N* = 105	*N* = 96	*N* = 155	*N* = 91
Admitted to hospital or hospice, n	9	24	23	37	39	41	62	17
Hospitalization	9 (100)	24 (100)	23 (100)	36 (97)	39 (100)	40 (98)	62 (100)	17 (100)
Hospice	0	1 (4)	0	2 (5)	0	2 (5)	0	0
Total no. hospitalizations^a^	13	47	39	87	77	78	161	22
No. hospitalizations per patient, median (range)	1 (1–3)	1 (1–6)	1 (1–5)	1 (0–13)	1 (1–7)	1 (0–8)	2 (1–9)	1 (1–2)
Total days of hospitalization per patient, mean (SD)	10.3 (6.4)	20.1 (23.0)	14.5 (14.5)	13.4 (12.0)	42.3 (26.4)	14.6 (13.1)	20.3 (20.4)	11.6 (9.3)
Median (range)	9 (3–21)	11 (2–88)	8 (1–49)	10 (2–54)	40 (3–108)	11 (1–56)	13 (2–104)	8 (2–30)
LOS per hospitalization (days)^b^								
Mean (SD)	7.2 (4.6)	10.5 (5.3)	8.5 (9.5)	5.5 (6.2)	21.7 (15.6)	7.5 (8.5)	7.8 (9.9)	9.0 (7.4)
Median (range)	8 (1–15)	10 (2–19)	5 (1–32)	4 (1–44)	17 (3–74)	5 (1–56)	4 (1–69)	7 (2–30)
No. hospitalizations per 100 pt-wk	0.60	3.57	2.76	3.63	5.13	3.93	3.47	1.59
Total no. emergency visits	6	52	0	30	6	41	82	25
No. emergency visits per 100 pt-wk	0.28	3.95	0	1.25	0.40	2.07	1.77	1.81
Patients with OP visits, n	72	50	28	96	60	79	90	40
OP visits, mean (SD)	6.9 (7.3)	6.1 (4.2)	5.3 (6.0)	7.3 (6.1)	9.6 (10.0)	9.1 (10.5)	15.4 (17.7)	4.2 (3.9)
OP visits, median (range)	4 (1–41)	6 (1–17)	3 (1–30)	5 (1–26)	5 (1–49)	5 (1–49)	10 (1–92)	3 (1–18)
No. OP visits per 100 pt-wk	23.00	23.26	10.40	29.08	38.23	36.29	29.79	12.18

^a^Hospitalizations did not include hospice stays

^b^For length of stay (LOS), extreme values at the upper end of the distribution were truncated at the 99th percentile of the study population

^c^Some of the hospitalization, emergency visit, and OP visit data for Japan have been previously reported by Isobe et al. [14]

*LOS* length of stay in days, *No.* number of, *OP* outpatient, *pt-wk* patient-weeks

**Fig. 3:**
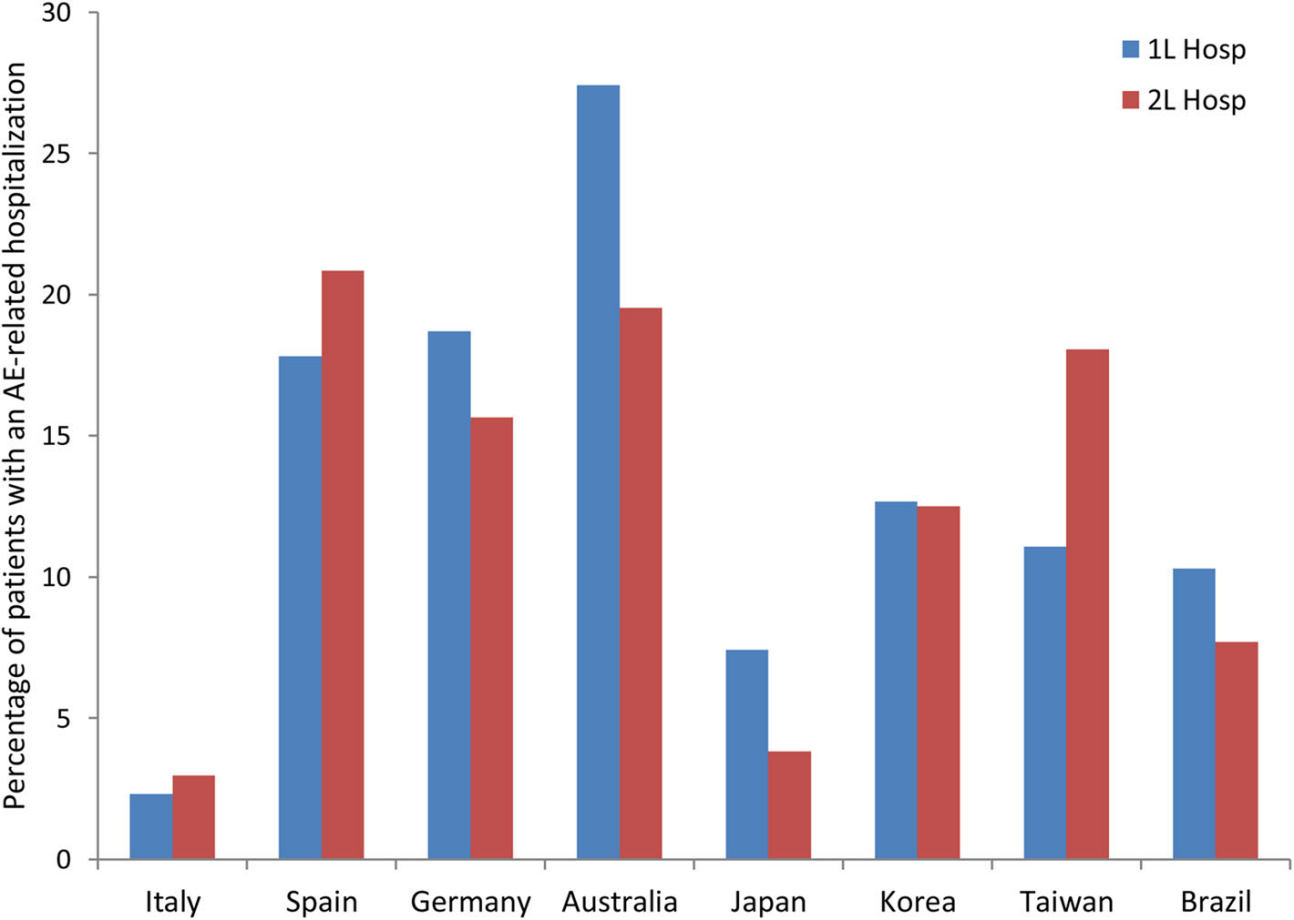
Hospitalizations resulting from a grade 3–5 AE during first- and second-line therapy. 1L, 2L, first- and second-line therapy; AE, adverse event

The percentages of patients who were hospitalized as the result of a grade 3–5 adverse event during first-line therapy were highest in Australia, followed by Germany and Spain; during second-line, the percentages were highest in Spain, Australia, and Taiwan (Fig. 3). In some countries a higher percentage was hospitalized in second-line than first-line (Spain, Taiwan, and Italy); in the other countries the reverse was true. Overall, the percentages who were hospitalized in both first- and second-line therapy because of a grade 3–5 adverse event were lowest in Italy, in line with overall hospitalizations, and were also relatively low in Japan.

**Fig. 4:**
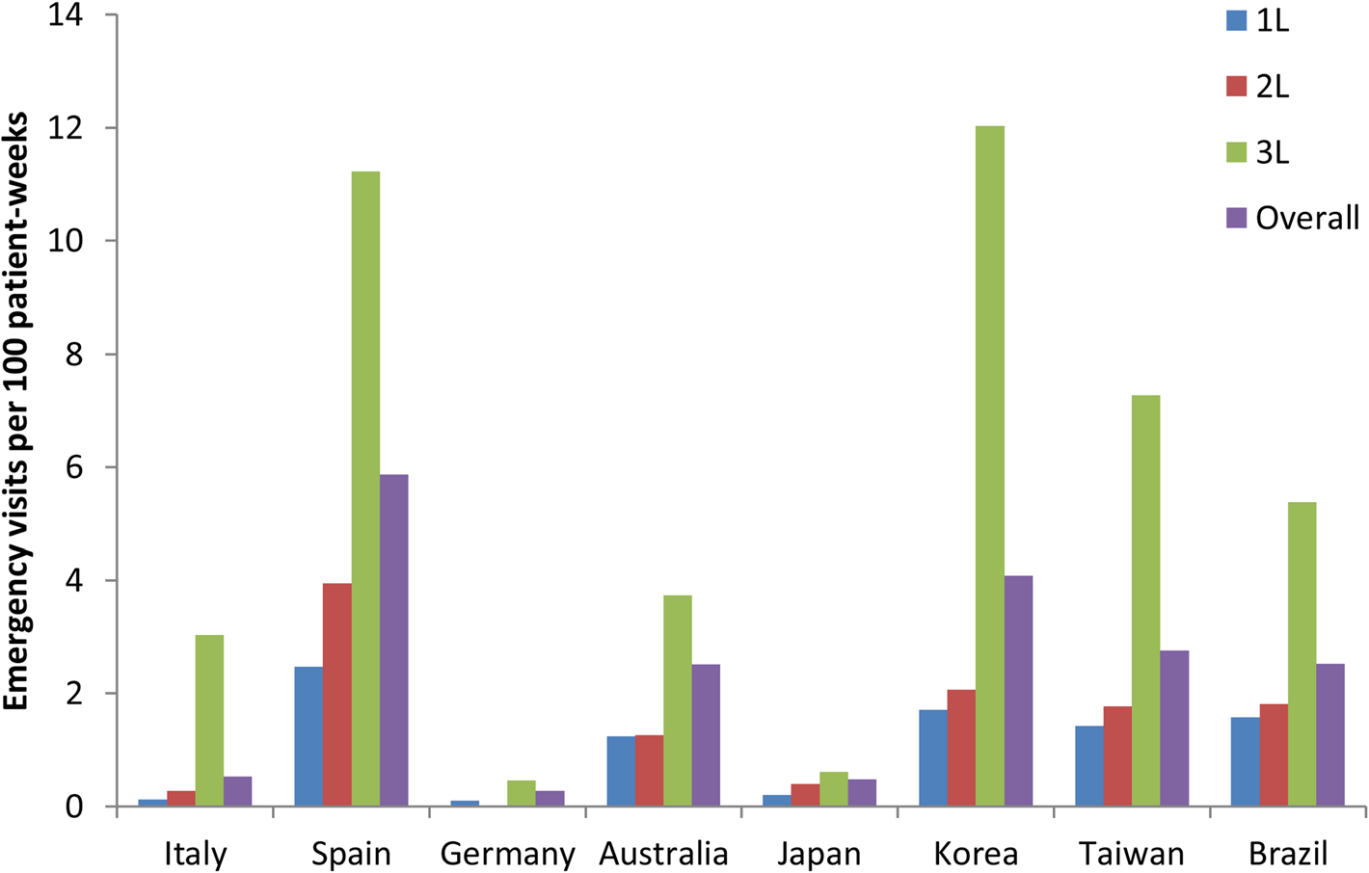
Number of emergency visits per 100 patient-weeks by line of therapy and overall in each country. 1L, 2L, 3L, during first-, second-, and third-line therapy

The frequency of emergency visits was low in Italy, Germany, and Japan and quite variable among the other countries (Table 3). However, in all countries a pattern of steadily increasing frequency from first- to third-line therapy was observed (Fig. 4).

**Fig. 5:**
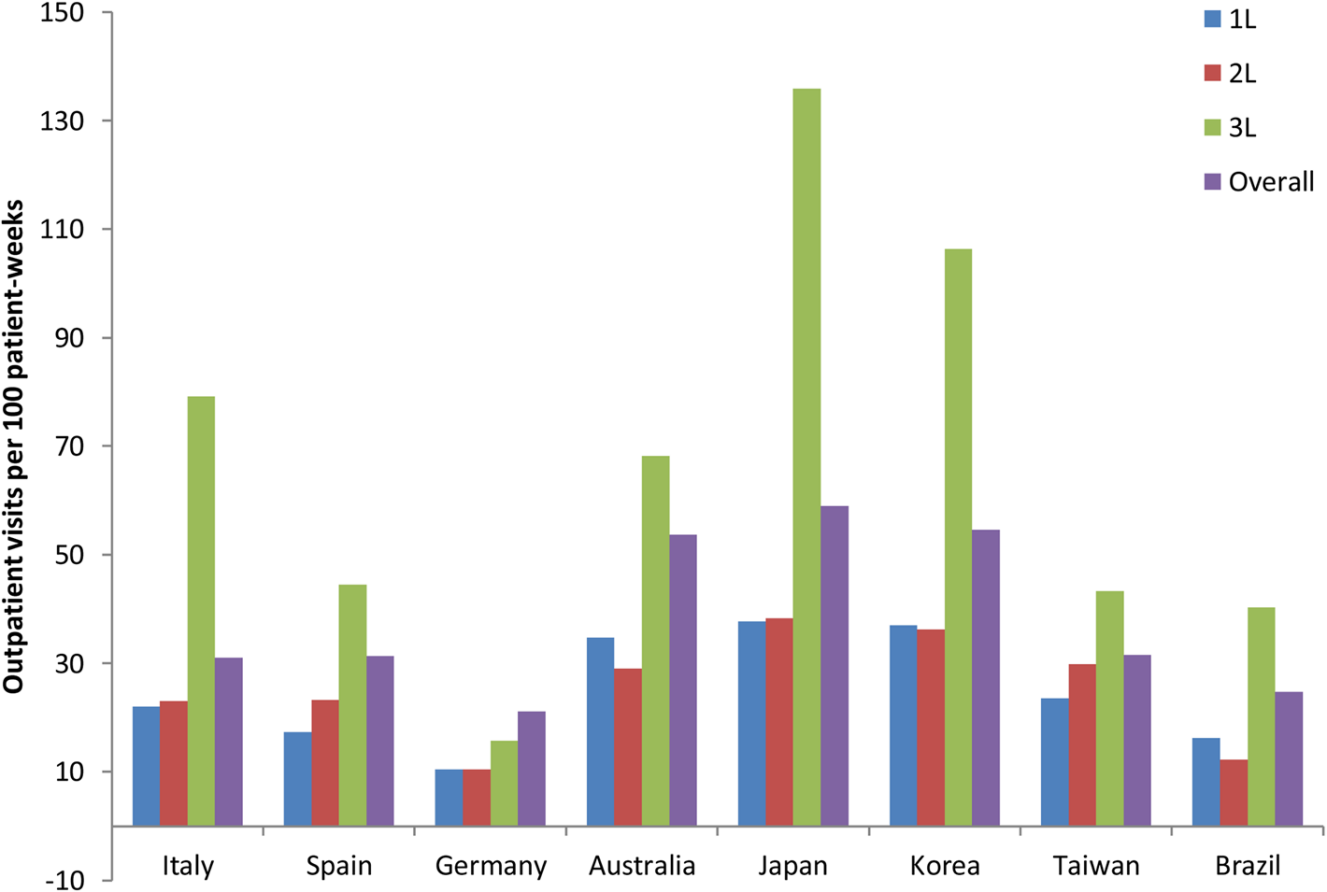
Number of outpatient visits per 100 patient-weeks by line of therapy and overall in each country. 1L, 2L, 3L, during first-, second-, and third-line therapy

The median number of outpatient visits per patient ranged from 4 (Germany) to 10 visits (Taiwan) during first-line therapy and from 3 (Germany and Brazil) to 10 visits (Taiwan) during second-line therapy. After accounting for follow-up time, the frequency of outpatient visits was greatest in Australia, Japan, and Korea during first-line (35–38 visits per 100 patient-weeks) and in Australia, Japan, Korea, and Taiwan during second-line (29–38 visits per 100 patient-weeks; Table 3, Fig. 5).

### Health care resource use by predictive biomarker status

Tables 4 and 5 depict key HCRU variables according to predictive biomarker testing status and the results of testing. Only in Germany were the comparative findings consistent during first- and second-line therapy: namely, the patients with positive *EGFR* mutation or *ALK* rearrangement status had longer median hospitalizations and more outpatient visits than those with negative or unknown test results.

**Table 4:** Resource use by predictive biomarker testing status in Italy, Spain, Germany, and Australia

	Italy (*N* = 174)			Spain (*N* = 202)			Germany (*N* = 139)			Australia (*N* = 208)		
	EGFR/ALK tested		Not tested	EGFR/ALK tested		Not tested	EGFR/ALK tested		Not tested	EGFR/ALK tested		Not tested
	Pos	Neg/Unk		Pos	Neg/Unk		Pos	Neg/Unk		Pos	Neg/Unk	
First-line therapy	*N* = 22	*N* = 66	*N* = 86	*N* = 24	*N* = 121	*N* = 57	*N* = 21	*N* = 55	*N* = 63	*N* = 31	*N* = 95	*N* = 82
No. inpatient stays^a^	13	26	36	13	72	28	17	49	74	40	209	78
Hospital	13 (100)	26 (100)	36 (100)	13 (100)	71 (99)	28 (100)	17 (100)	49 (100)	74 (100)	35 (88)	175 (84)	74 (95)
Hospice	0	0	0	0	1 (1)	0	0	0	0	5 (13)	34 (16)	4 (5)
No. hospitalizations^b^	13	26	36	13	71	28	15	52	85	35	177	74
Normal ward	13 (100)	26 (100)	35 (97)	13 (100)	71 (100)	28 (100)	15 (100)	48 (92)	72 (85)	35 (100)	175 (99)	74 (100)
ICU	0	0	1 (3)	0	0	0	0	4 (8)	13 (15)	0	2 (1)	0
No. hospitalizations with LOS^c^	13	25	35	13	70	28	17	49	74	35	173	73
Mean (SD) days	4.5 (4.2)	7.8 (10.0)	5.4 (6.9)	7.5 (6.4)	8.8 (5.8)	9.1 (5.9)	13.6 (12.4)	9.7 (12.3)	7.4 (10.1)	3.5 (5.6)	4.6 (4.8)	5.2 (7.5)
Median (range)	4 (1–14)	5 (3–50)	1 (1–26)	6 (2–27)	7 (1–28)	7 (1–26)	9 (1–32)	4 (1–32)	3 (1–32)	1 (1–33)	3 (1–27)	3 (1–48)
Outpatient visits, *n*^d^	*19*	*63*	*64*	*21*	*92*	*32*	*11*	*25*	*32*	*29*	*93*	*73*
Mean (SD) visits	9.1 (8.4)	8.9 (8.3)	5.7 (3.8)	9.4 (9.7)	11.8 (12.8)	7.8 (7.1)	16.0 (10.7)	10.6 (9.7)	7.6 (9.7)	26.7 (30.3)	12.9 (12.4)	7.8 (6.9)
Median (range)	8 (1–35)	6 (1–34)	5 (1–17)	6 (1–42)	9 (1–58)	6 (1–23)	13 (2–31)	7 (1–41)	3 (1–33)	21 (1–105)	9 (1–57)	6 (1–33)
No. outpatient visits	173	562	367	198	1089	250	176	266	242	774	1203	569
Second-line therapy	*N* = 14	*N* = 41	*N* = 46	N = 14	*N* = 62	N = 20	*N* = 14	*N* = 27	*N* = 23	*N* = 22	*N* = 66	*N* = 40
No. inpatient stays^a^	3	5	5	10	33	6	14	13	12	20	51	18
Hospital	3 (100)	5 (100)	5 (100)	8 (80)	33 (100)	6 (100)	14 (100)	13 (100)	12 (100)	20 (100)	49 (96)	18 (100)
Hospice	0	0	0	2 (20)	0	0	0	0	0	0	2 (4)	0
No. hospitalizations^b^	3	5	5	8	33	6	14	13	12	20	49	18
Normal ward	3 (100)	5 (100)	5 (100)	8 (100)	33 (100)	6 (100)	14 (100)	13 (100)	12 (100)	20 (100)	49 (100)	18 (100)
ICU	0	0	0	0	0	0	0	0	0	0	0	0
No. hospitalizations with LOS^c^	3	5	5	8	32	6	14	13	12	20	49	18
Mean (SD) days	8.3 (1.2)	9.8 (4.3)	3.8 (4.4)	12.3 (5.3)	10.2 (5.4)	10.0 (4.5)	10.3 (10.5)	7.0 (11.1)	8.2 (6.1)	4.7 (3.4)	5.6 (7.4)	6.3 (4.7)
Median (range)	9 (7–9)	9 (4–15)	1 (1–11)	14 (5–17)	10 (3–19)	11 (2–15)	6 (1–32)	1 (1–32)	8 (1–19)	4 (1–9)	3 (1–44)	6 (2–21)
Outpatient visits, *n*^d^	13	34	25	9	41	12	12	13	10	18	62	22
Mean (SD) visits	11.5 (12.6)	5.8 (5.9)	6.1 (3.8)	8.3 (4.2)	6.6 (4.8)	7.0 (3.9)	8.2 (10.4)	4.7 (4.2)	6.1 (4.4)	7.3 (5.3)	8.6 (7.1)	5.2 (4.3)
Median (range)	6 (1–41)	3 (1–25)	6 (1–16)	7 (3–15)	6 (1–17)	8 (1–12)	4 (1–30)	2 (1–12)	7 (1–11)	6 (1–20)	6 (1–26)	4 (1–17)
No. outpatient visits	149	196	153	75	269	84	98	61	61	131	533	115

^a^Total number of inpatient stays, defined as one or more nights in a hospital or hospice

^b^Total number of hospitalizations, not including hospice stays

^c^Total number of hospitalizations with non-missing data regarding length of stay (LOS)

^d^Number of patients who had ≥1 outpatient visits

*ALK* anaplastic lymphoma kinase, *EGFR* epidermal growth factor receptor, *LOS* length of stay, *Neg/Unk* negative/unknown, *No.* number of, *OP* outpatient, *Pos* positive

**Table 5:** Resource use by predictive biomarker testing status in Japan, Korea, Taiwan, and Brazil

	Japan (*N* = 175)			Korea (*N* = 150)			Taiwan (*N* = 217)			Brazil (*N* = 175)		
	EGFR/ALK tested		Not tested	EGFR/ALK tested		Not tested	EGFR/ALK tested		Not tested	EGFR/ALK tested		Not tested
	Pos	Neg/Unk		Pos	Neg/Unk		Pos	Neg/Unk		Pos	Neg/Unk	
First-line therapy	*N* = 49	*N* = 81	*N* = 45	*N* = 53	*N* = 59	*N* = 38	*N* = 126	*N* = 59	*N* = 32	*N* = 13	*N* = 61	*N* = 101
No. inpatient stays^a^	44	129	69	89	82	80	90	83	27	5	17	20
Hospital	44 (100)	129 (100)	69 (100)	89 (100)	82 (100)	80 (100)	90 (100)	83 (100)	27 (100)	5 (100)	17 (100)	20 (100)
No. hospitalizations^b^	44	131	69	89	82	80	90	83	29	5	19	20
Normal ward	44 (100)	129 (99)	69 (100)	89 (100)	82 (100)	80 (100)	90 (100)	83 (100)	27 (93)	4 (80)	17 (90)	20 (100)
ICU	0	2 (2)	0	0	0	0	0	0	2 (7)	1 (20)	2 (11)	0
No. hospitalizations with LOS^c^	43	125	68	89	82	78	89	83	27	5	16	20
Mean (SD) days	25.3 (16.9)	18.9 (15.4)	15.5 (12.8)	6.7 (7.4)	7.7 (11.1)	6.8 (9.9)	12.9 (14.7)	4.7 (3.3)	8.6 (8.6)	30.8 (24.2)	17.8 (25.7)	12.9 (14.8)
Median (range)	21 (2–69)	13 (1–81)	13 (2–62)	3 (1–39)	3 (1–47)	3 (1–45)	7 (1–71)	3 (1–17)	5 (2–31)	22 (4–68)	6 (1–77)	6 (2–55)
Outpatient visits, *n*^d^	*34*	*58*	*33*	*75*	*80*	*41*	*81*	*44*	*24*	*8*	*41*	*49*
Mean (SD) visits	17.5 (13.0)	14.9 (23.7)	16.5 (18.4)	11.8 (11.0)	14.2 (14.5)	11.3 (6.9)	16.3 (17.1)	8.0 (7.0)	17.0 (17.4)	8.3 (7.5)	9.5 (7.7)	5.1 (5.9)
Median (range)	15 (1–51)	7 (1–124)	8 (1–62)	9 (1–51)	9 (1–58)	11 (1–27)	12 (1–109)	6 (1–27)	12 (2–76)	6 (2–22)	8 (1–35)	4 (1–36)
No. outpatient visits	595	863	546	882	1132	465	1321	352	407	66	389	251
Second-line therapy	*N* = 32	*N* = 46	*N* = 27	*N* = 37	*N* = 37	*N* = 22	*N* = 85	*N* = 44	*N* = 26	*N* = 8	*N* = 42	*N* = 41
No. inpatient stays^a^	26	27	24	29	18	32	120	28	13	1	11	10
Hospital	26 (100)	27 (100)	24 (100)	29 (100)	18 (100)	31 (97)	120 (100)	28 (100)	13 (100)	1 (100)	11 (100)	10 (100)
Hospice	0	0	0	0	0	1 (3)	0	0	0	0	0	0
No. hospitalizations^b^	26	27	24	29	18	33	120	28	13	1	11	10
Normal ward	26 (100)	27 (100)	24 (100)	29 (100)	17 (94)	31 (94)	120 (100)	28 (100)	13 (100)	1 (100)	11 (100)	10 (100)
ICU	0	0	0	0	1 (6)	2 (6)	0	0	0	0	0	0
No. hospitalizations with LOS^c^	26	27	23	29	18	31	120	28	13	1	11	10
Mean (SD) days	21.7 (15.0)	21.5 (16.2)	22.0 (16.4)	8.7 (11.5)	8.2 (7.1)	5.9 (5.4)	6.3 (7.4)	12.5 (15.7)	12.2 (10.5)	5.0 (n/a)	6.7 (5.3)	11.8 (8.8)
Median (range)	17 (3–63)	17 (3–74)	15 (4–54)	4 (1–56)	8 (1–26)	2 (1–18)	3 (1–52)	6 (3–69)	9 (3–37)	5	6 (2–21)	9 (2–30)
Outpatient visits, *n*^d^	*18*	*26*	*19*	*48*	*41*	*18*	*45*	*27*	*18*	*2*	*22*	*18*
Mean (SD) visits	6.8 (7.8)	7.9 (6.6)	16.1 (13.8)	14.8 (14.0)	8.6 (7.2)	9.6 (13.2)	11.7 (11.4)	19.9 (23.6)	17.8 (19.7)	3.0 (0)	5.7 (4.9)	2.9 (2.0)
Median (range)	3 (1–24)	6 (1–26)	14 (2–49)	8 (1–49)	5 (1–21)	5 (1–45)	9 (1–55)	12 (1–92)	12 (1–68)	3 (3–3)	5 (1–18)	3 (1–6)
No. outpatient visits	122	205	305	710	351	172	525	537	320	6	126	53

^a^Total number of inpatient stays, defined as one or more nights in a hospital or hospice. There were no hospice stays in Japan, Korea, Taiwan, or Brazil in association with first-line therapy

^b^Total number of hospitalizations, not including hospice stays

^c^Total number of hospitalizations with non-missing data regarding length of stay (LOS)

^d^Number of patients who had ≥1 outpatient visits

*ALK* anaplastic lymphoma kinase, *EGFR* epidermal growth factor receptor, *LOS* length of stay, *Neg/Unk* negative/unknown, *No*. number of, *OP* outpatient, *Pos* positive

In the other countries, there was no consistency in the pattern or differences in HCRU by biomarker status between the first and second lines of therapy. For example, during first-line therapy in Taiwan, which had the largest *EGFR/ALK*-positive cohort (*n* = 126), patients with positive *EGFR* mutation or *ALK* rearrangement status had longer median hospitalizations (7 vs. 3 days) and more outpatient visits (median, 12 vs. 6 visits) than those with negative or unknown test status, whereas during second-line therapy, the inverse was true (median 3 vs. 6 days in hospital and median of 9 vs. 12 outpatient visits, respectively; Table 5). In Korea, the median hospitalization lengths and median number of outpatient visits were the same or similar whether patients had positive or negative *EGFR/ALK* mutation status or had not been tested.

### Health care resource use associated with procedures

The median number of imaging tests per patient in association with first-line therapy was greatest in Japan (14 imaging tests), followed by Taiwan (median of 8) and Korea (median of 7; Table 6). The number of imaging tests per patient fell from first-line to second-line in all countries except Korea, where the median number of imaging tests per patient increased from 7 during first-line therapy to 8 during second-line therapy. Korea was also the only country in which positron emission tomography (PET) scans were frequently used: 70% of patients in Korea had a PET scan during first-line as compared with one-third or fewer patients in the other countries. Computed tomography (CT) scans were the most commonly employed imaging test in all countries, with 89% or more of patients in each country receiving a CT scan in association with first-line therapy (Table 6).

**Table 6:** Health care resource use associated with procedures

	Italy		Spain		Germany		Australia		Japan		Korea		Taiwan		Brazil	
	1LT	2LT	1LT	2LT	1LT	2LT	1LT	2LT	1LT	2LT	1LT	2LT	1LT	2LT	1LT	2LT
	*N* = 174	*N* = 101	*N* = 202	*N* = 96	*N* = 139	*N* = 64	*N* = 208	*N* = 128	*N* = 175	*N* = 105	*N* = 150	*N* = 96	*N* = 217	*N* = 155	*N* = 175	*N* = 91
No. imaging tests, n	155	85	186	72	133	53	191	115	160	97	142	91	192	117	149	72
Mean (SD)	3.4 (2.6)	3.0 (3.1)	4.6 (3.4)	3.9 (3.3)	7.0 (4.9)	5.3 (3.4)	5.4 (5.0)	4.7 (4.3)	16.7 (12.7)	10.2 (7.8)	9.3 (8.7)	10.0 (8.9)	11.5 (13.4)	10.6 (14.8)	3.6 (3.7)	3.0 (2.4)
Median (range)	3 (1–13)	2 (1–17)	4 (1–23)	3 (1–21)	6 (1–31)	5 (1–16)	4 (1–33)	3 (1–23)	14 (1–68)	8 (1–39)	7 (1–75)	8 (1–48)	8 (1–86)	5 (1–86)	3 (1–31)	2 (1–11)
Imaging test type, n (%)^a^																
Radiograph	42 (27)	16 (19)	77 (41)	25 (35)	93 (70)	30 (57)	112 (59)	55 (48)	147 (92)	87 (90)	99 (70)	68 (75)	123 (64)	87 (74)	16 (11)	7 (10)
PET scan	45 (29)	12 (14)	39 (21)	5 (7)	41 (31)	5 (9)	56 (29)	7 (6)	14 (9)	4 (4)	99 (70)	22 (24)	42 (22)	1 (1)	43 (29)	6 (8)
CT scan	146 (94)	78 (92)	173 (93)	65 (90)	127 (96)	48 (91)	170 (89)	109 (95)	145 (91)	88 (91)	131 (92)	90 (99)	180 (94)	86 (74)	136 (91)	69 (96)
MRI	28 (18)	10 (12)	70 (38)	21 (29)	56 (42)	12 (23)	32 (17)	20 (17)	107 (67)	58 (60)	103 (73)	33 (36)	72 (38)	32 (27)	52 (35)	15 (21)
Ultrasound	8 (5)	6 (7)	18 (10)	2 (3)	19 (14)	2 (4)	46 (24)	17 (15)	19 (12)	2 (2)	25 (18)	5 (6)	39 (20)	19 (16)	8 (5)	5 (7)
Other	16 (10)	4 (5)	32 (17)	10 (14)	61 (46)	9 (17)	38 (20)	15 (13)	92 (58)	31 (32)	53 (37)	29 (32)	102 (53)	26 (22)	48 (32)	11 (15)
No. biopsy-related procedure	129	1	147	3	124	3	146	1	147	0	129	3	168	2	113	1
Mean (SD)	1.0 (0.2)	1 (n/a)	1.1 (0.3)	1.0 (0)	1.2 (0.4)	1.0 (0)	1.1 (0.3)	1.0 (n/a)	1.1 (0.4)	n/a	1.2 (0.4)	1.3 (0.6)	1.1 (0.2)	1.0 (0)	1.0 (0.2)	n/a
Median (range)	1 (1–2)	1	1 (1–3)	1 (1–1)	1 (1–4)	1 (1–1)	1 (1–3)	1 (1–1)	1 (1–5)	n/a	1 (1–2)	1 (1–2)	1 (1–2)	1 (1–1)	1 (1–2)	1 (1–1)
No. biomarker tests	67	6	120	10	67	3	89	16	84	2	102	6	150	3	45	11
Mean (SD)	1.2 (0.4)	1 (0)	1.5 (0.9)	1.0 (0)	2.4 (1.4)	1.7 (1.2)	1.5 (0.8)	1.7 (1.1)	1.1 (0.4)	1.0 (0)	2.2 (0.9)	1.7 (1.2)	1.0 (0)	1.0 (0)	1.1 (0.3)	1.2 (0.4)
Median (range)	1 (1–2)	1 (1–1)	1 (1–6)	1 (1–1)	2 (1–8)	1 (1–3)	1 (1–3)	1 (1–5)	1 (1–2)	1 (1–1)	2 (1–4)	1 (1–4)	1 (1–1)	1 (1–1)	1 (1–2)	1 (1–2)
Test type, number																
ALK	17	4	30	1	34	2	16	8	14	1	48	2	0	0	7	4
EGFR	64	2	110	8	62	1	85	10	81	1	96	5	150	3	42	8
Other	2	0	18	1	47	2	29	7	0	0	80	2	0	0	0	1

^a^In Germany, the imaging test type was unknown or missing for two imaging tests during first-line and one imaging test during second-line therapy

*1LT* first-line therapy, *2LT* second-line therapy, *ALK* anaplastic lymphoma kinase, *CT* computed tomography, *EGFR* epidermal growth factor receptor, *LOS* length of stay, *MRI* magnetic resonance imaging, *n/a* not applicable, *No*. number of, *OP* outpatient, *PET* positron emission tomography

Biopsy-related procedures were employed almost exclusively in association with first-line therapy (Table 6). Similarly, predictive biomarker tests were run most frequently in association with first-line therapy, although some patients were tested for biomarkers in second-line, particularly in Australia. The most common biomarker testing in all countries was for *EGFR* mutations, with testing for *ALK* rearrangements second in frequency. In Germany, Australia, and Korea, other tests such as for activating KRAS mutations were run for a substantial number of patients (Table 6).

## Discussion

This retrospective observational study documented the health care resource use (HCRU) associated with first and subsequent lines of systemic therapy for advanced NSCLC for 1440 patients in eight countries. We found substantial regional variation in HCRU parameters, including the frequency and length of hospitalizations and the frequency of outpatient visits. Hospitalizations were most frequent in Japan, Korea, Germany, and Australia, relative to the other countries, and the length of each hospital stay was longest in Japan during each line of therapy. While the frequency of emergency visits also varied among countries, we detected a consistent increase in the frequency of emergency visits from first- through third-line therapy in each country. The outpatient setting was the most common setting of resource use. The evaluation of HCRU findings by predictive biomarker testing status identified no regular pattern, and the utilization and selection of imaging tests and predictive biomarker tests also varied among the countries. These findings suggest differing approaches to the clinical management of advanced NSCLC in the eight countries in this study.

There are few prior studies of real-world HCRU with which to compare our findings, in part because of differences in patient populations and in assessed variables. We conducted this study using one standard protocol in these eight countries in different regions in order to benchmark contemporary treatment practices and HCRU for patients receiving systemic treatment for advanced NSCLC. Nonetheless, our findings are purely descriptive and, because of the differences across countries in patient populations and health care settings, we cannot make definitive comparative statements. A 2013 review identified only two international studies of treatment patterns for NSCLC [10], neither of which reported HCRU findings [15, 16]. More recently, two multinational observational studies, both prospective, have been conducted in Europe, one limited to patients in 11 countries with advanced NSCLC prescribed first-line platinum-based chemotherapy (FRAME [17]) and the other including patients in 8 countries with all stages of NSCLC (EPICLIN-Lung [18]), thus neither directly comparable to our study. The HCRU variables measured in these two studies also differed from those we used, and most results were pooled for the participating countries.

The few HCRU findings from FRAME and EPICLIN-Lung that can be compared with ours relate to hospitalizations. In FRAME, 55% of patients were hospitalized during first-line therapy, a percentage that ranged in the present study from 24% in Italy, to 42% in Spain, to 75% in Germany [17]. In EPICLIN-Lung, the total median numbers of hospital days for each patient were 9 and 10 days for stage IIIB and stage IV, respectively, as compared with medians of 8–13 days during first-line and 8–11 days during second-line therapy in the European countries in our study [18].

Our finding of the longest hospital stays being in Japan (mean of 19.1 and 21.7 days in first- and second-line, respectively) is consistent with results of the Organisation for Economic Cooperation and Development (OECD) summary of length of hospital stay for acute care (all causes) in 2015, in which the mean hospital stay for Japan (16.5 days) was the outlier among member countries, with Korea having the next longest mean stay (8.0 days) [19]. Few other studies have reported on hospitalization rates in individual countries. In one Australian medical center, patients with stage III and IV NSCLC spent a median of 13 and 18 total days in hospital, respectively [20], as compared with medians of 9 plus 10 total days during first- plus second-line, respectively, in our Australian cohort. In Canada, the frequency of hospitalizations was 7.0 per 100 patient-weeks after the completion of chemotherapy [21], as compared with the overall frequency in our study of 2.5–11.1 per 100 patient-weeks, depending on country.

Patients with advanced NSCLC may be hospitalized for different reasons and at different times during the course of their illness. Reasons for hospitalization include initial and subsequent diagnostic testing, anticancer therapy administration, patient monitoring, adverse event management, and provision of supportive care. The decision to hospitalize a patient in any given situation is likely influenced by preferred clinical practices, which could explain the variability in frequency and length of hospitalizations found in this study. The pattern of hospitalizations resulting from grade 3–5 adverse events did not follow the overall pattern of hospitalizations (for any reason), further suggesting that clinical practices varied among the countries. The percentages of patients who were hospitalized because of a grade 3–5 adverse event during first-line therapy were highest in Australia and lowest in Italy, in line with overall hospitalizations, but were also relatively low in Japan, where overall hospitalizations were most frequent. Clinical trials rarely report information on hospitalizations, and the results of one recent review concluded that real-world patients undergoing chemotherapy for metastatic NSCLC have significantly higher rates of hospitalization than those in clinical trials receiving similar therapy [11], further supporting the need for real-world studies such as this one.

In European countries, lung cancer had the greatest associated economic burden of four common cancers (including also breast, colorectal, and prostate cancer) [22]. We did not evaluate the costs associated with HCRU in the present study; however, hospitalizations are acknowledged as being a major cost driver for patients with advanced NSCLC in studies in several countries [20, 21, 23–26]. Other important factors driving costs include fees for medical specialists in the Netherlands [23], physicians’ fees [25] and outpatient and inpatient medical services [27] in the United States, and drug costs in China, where drug consumption is a means of paying for physicians’ work [28].

One of the strengths of our study is that we were able to examine HCRU findings by predictive biomarker testing status. We found no regular pattern of HCRU in association with first- or second-line therapy according to whether patients were tested for *EGFR* mutation or *ALK* rearrangement, nor whether the results were positive, negative, or unknown. In Taiwan, where testing for *EGFR* mutation was most frequent and first-line therapy with an EGFR TKI was most common, the relatively large cohort of patients with positive *EGFR/ALK* status experienced longer hospitalizations and more outpatient visits during first-line therapy but shorter hospitalizations and fewer outpatient visits during second-line therapy than patients whose EGFR/ALK test results were negative or unknown. However, the proportion of patients who received EGFR/ALK TKIs was much lower in second-line as compared with first-line therapy in Taiwan, hence the *EGFR/ALK* status may not have had an impact on resource use. These findings require further investigation. Results of an earlier small study in Korea suggested that the mean monthly costs were lower for patients with *EGFR*-mutation positive status who received targeted treatment for advanced NSCLC as compared with patients who had wild-type EGFR status [29].

The frequency of emergency visits increased in all countries in this study over time, from first- to third-line therapy. Lung cancer, as compared with other cancers, has been identified in a recent systematic review as being a risk factor for emergency department attendance in the last month of life [30]. The authors of this review did not offer an explanation for their finding but note that future investigations are needed of psychosocial factors and patient and caregiver preferences for using emergency care services. The use of health care resources is driven by clinicians’ and patients’ preferences, and of course by patients’ performance status and the presence or absence of metastases and comorbidities. As previously reported, hospitalizations and HCRU increase with skeletal metastases [31–33], and patients with brain metastases have greater HCRU and incur greater health care costs [34–36].

Regional and within-country variations in NSCLC treatment patterns and HCRU parameters have been recorded also in prior studies. We can speculate that these differences are at least in part attributable to variability in NSCLC management guidelines and in health care systems, including reimbursement policies and insurance options. The median age of patients varied among countries from 63 to 70 years, and the Taiwanese study population was notable in having the lowest proportion of men and current or former smokers, perhaps because of variability in attendance at study sites or possible selection bias. Tsukada and coworkers [37] studied the characteristics of cancer populations among different types of hospitals in Japan, and they found higher proportions of early-stage cancer and younger patients at higher-volume hospitals than at lower-volume hospitals. Substantial variations in patterns of care for NSCLC among the different hospital types were recorded in their study in Japan [37], as well as in prior observational studies in Taiwan [38] and the Netherlands [39, 40]. Our study included a mix of hospital types. All sites in Australia and Taiwan, and the majority of sites in Spain (10 of 13 [10/13]) and Korea (2/3) were centers affiliated with an academic institution; in Italy, Germany, and Japan the inverse was true, as 3/9, 3/10, and 1/5 sites, respectively, were academic; and in Brazil, study sites were a mix of academic (*n* = 6) and non-academic (*n* = 5) [13]. Therefore, within-country variation in patterns of care among different sites in our study is also possible.

A limitation of this study is that we were not able to link patient and NSCLC clinical characteristics with the quantity of HCRU. Other study limitations are those inherent to observational studies using medical records, including the afore-mentioned potential for selection bias in addition to the possibility of missing and/or incorrectly recorded data. A standardized approach was used to collect study data at each study site using the eCRFs, and we provided training to the site coordinators to promote consistent recording among the study sites; however, the potential for inconsistent recording remains a study limitation.

Strengths of this descriptive study include the large patient population and the capture of real-world resource use for systemic therapy of advanced NSCLC in eight different countries. We evaluated and report HCRU according to line of therapy, including three lines of therapy, and overall, in addition to biomarker testing status, with a minimum of 12 months’ follow-up for each patient. The diagnosis of advanced NSCLC occurred from the start of 2011 to mid-2014; thus, the data illustrate relatively recent clinical practices for the diagnosis and treatment of advanced NSCLC.

Further research is needed to evaluate HCRU in countries not included in this study, as well as to update and expand our findings in the countries studied, to inform the administration of health care resources. A contemporary comparison of HCRU for NSCLC among different hospital types within each country is an important topic for future study that was beyond the scope of the present study. Another important topic for future study is to identify the proportion of patients who do not receive treatment for advanced NSCLC and the reasons why treatment is not provided to (or chosen by) patients.

## Conclusions

In 2013, De Geer et al. [10] noted the scarcity of studies of real-world treatment patterns and HCRU for advanced NSCLC. This remains largely true today. The prevalence of lung cancer has decreased slightly among men in some countries but is projected to continue rising among both men and overall in other countries, particularly where tobacco smoking remains common [1]. Lung cancer will remain a major health care burden for the foreseeable future. We hope that the data provided here will be useful for policy- and decision-makers in each country to determine future resource allocation. More studies are needed of real-world clinical practices and HCRU for advanced NSCLC to benchmark current practices as a baseline in light of the ever-increasing availability of new therapies, associated resource-intensive health care needs, and increasing budgetary pressures on health care systems. Comparative findings and an understanding of country-specific clinical practices are essential to identify areas of need and to guide future resource allocation for patients with advanced NSCLC.

## Additional file

Additional file 1: Ethics Committee Names and Locations. (XLSX 13 kb)

## Abbreviations

ALK: Anaplastic lymphoma kinase
CT: Computed tomography
eCRF: Electronic case report forms
EGFR: Epidermal growth factor receptor
HCRU: Health care resource use
LOT: Line of therapy
NSCLC: Non-small cell lung cancer
PD-1: Programmed death-1
PET: Positron emission tomography
TKI: Tyrosine kinase inhibitor
